# Case report: *Acinetobacter baumannii* septic arthritis in an immunocompetent infant

**DOI:** 10.3389/fmed.2023.1135178

**Published:** 2023-03-01

**Authors:** Yi Liao, Jiapeng Xiao, Feng Fang, Hua Zhou, Lingling Liu, Xinglou Liu

**Affiliations:** ^1^Department of Pediatrics, Tongji Hospital, Tongji Medical College, Huazhong University of Science and Technology, Wuhan, China; ^2^Department of Pediatrics, Luotian County People's Hospital, Huanggang, China

**Keywords:** *Acinetobacter baumannii*, septic arthritis, infant, metagenomics next-generation sequencing, case report

## Abstract

*Acinetobacter baumannii* is a gram-negative coccobacilli, mainly causing nosocomial infections with poor prognosis, especially in patients with prolonged hospitalization or antibiotics administration. *A. baumannii* pneumonia is the most common clinical form and usually occurs in critically ill patients in the intensive care unit. However, septic arthritis caused by *A. baumannii* is rarely reported. In this report, we describe a case of *A. baumannii* septic arthritis combined with incomplete Kawasaki disease in an infant. The child's chief complaint was a 2-week intermittent fever with poor response to antibiotics. Initial physical examination revealed swollen lymph nodes in the neck, pharynx congestion, and the appearance of rashes. Combined with laboratory tests, the diagnosis of incomplete Kawasaki disease was considered. After administration of high-dose intravenous immunoglobulin and corticosteroids, the child's fever improved and periungual desquamation appeared simultaneously. Swelling of the right knee occurred 5 days after the fever improved and imaging tests of MRI and ultrasound suggested the existence of infection. A diagnosis of septic arthritis was established subsequently, and arthroscopy was carried out. *A. baumannii* was finally identified by metagenomics next-generation sequencing of joint draining fluid for pathogenic microorganisms. Treatment with meropenem was then started. The patient eventually recovered and was discharged from the hospital after 23 days of treatment with meropenem. Although *A. baumannii* is not a common bacterium of septic arthritis, this rare infection can still occur in infants. Early diagnosis, pathogenic identification, and target antibiotic treatment are important to reduce the occurrence of joint sequelae.

## Introduction

*Acinetobacter baumannii* is gram-negative, non-motile, aerobic coccobacilli, and is the most important member of the *Acinetobacter* species associated with hospital-acquired infections (1). As an opportunistic pathogen, *A. baumannii* usually affects immunocompromised individuals, particularly those with a long history of hospitalization or antibiotics administration, causing meningitis, pneumonia, endocarditis, wound infections, and infections of the bloodstream, urinary tract, skin, and other soft tissues (2). In recent years, *A. baumannii* has been reported to rapidly develop resistance to antimicrobials. Multidrug-resistant strains, and even pandrug-resistant (3) strains have been isolated, leading to a high mortality rate after infection, which has become a major concern for scientists (2, 4). The most common nosocomial *A. baumannii* infection is pneumonia, mainly occurring in patients staying in an intensive care unit and breathing through a ventilator (2, 5). However, septic arthritis (SA) caused by *A. baumannii* is a rare condition, and to date, seldom reported in adult patients with puncture, surgery, or penetrating wounds of the knee joint (6–8). In this report, we describe a case of *A. baumannii* SA combined with incomplete Kawasaki disease (KD) in an immunocompetent child, which may help pediatric professionals to further understand *A. baumannii* and provide new clues for clinical diagnosis and treatment.

## Case description

A 10-month-old boy was admitted to our hospital for 2 weeks with intermittent fever. His disease started with a low-grade fever, which peaked at 40°C the next day, occurring one to three times per day. Then, the patient visited the local hospital, and laboratory findings showed a white blood cell (WBC) count of 7.75 × 10^9^/L (reference range: 5.00–15.00 × 10^9^/L), with a neutrophils percentage of 30.6% (reference range: 10.0–56.9%). Hemoglobin and platelet levels were 11.8 g/dL (reference range: 10.0–14.0 g/dL) and 280 × 10^9^/L (reference range: 150–450 × 10^9^/L), respectively. The C-reactive protein (CRP) level was normal. After being given symptomatic treatment, the boy had no fever for 2 days. However, the fever reappeared subsequently, accompanied by scattered red rashes all over the body, diarrhea, and a mild cough. Full blood count (FBC) testing was then repeated, and the WBC count was 5.22 × 10^9^/L with a neutrophil ratio of 60.6%. The CRP level increased to 81.3 mg/L (reference range: <0.5 mg/L) and the procalcitonin level was also significantly elevated (8.02 ng/ml, reference range: <0.05 ng/ml), indicating severe infection. Alanine aminotransferase and aspartate aminotransferase levels were 58 (reference range: ≤41 U/L) and 76 U/L (reference range: ≤40 U/L), respectively, suggesting a mild liver injury. The patient was treated with intravenous antibiotics (mezlocillin sodium, 100 mg/kg/d and cefoperazone/ sulbactam sodium, 100 mg/kg/d), corticosteroid, and intravenous immunoglobulin (IVIG) (0.25 g/kg/day over 3 days). After the aforementioned treatments were given, the levels of CRP and procalcitonin returned almost to normal, but the boy's temperature was not well-controlled.

For further diagnosis and treatment, the boy was admitted to our hospital 2 weeks after the onset of symptoms. An initial physical examination revealed small red rashes scattered throughout the body, pharynx congestion, and swollen lymph nodes in the neck. Laboratory investigations were performed subsequently. FBC testing showed leukocytosis (WBC count of 24.02 × 10^9^/L with a neutrophil ratio of 48.5%), anemia (hemoglobin 8.9 g/dL), and thrombocytosis (platelet count of 507 × 10^9^/L). The CRP (65.02 mg/L) and procalcitonin (1.99 ng/ml) levels were elevated once more. Hypoalbuminemia (albumin 30.0 g/L, reference range: 38.0–54.0 g/L) and a high erythrocyte sedimentation rate (ESR, 57 mm/h, reference range: 0–15 mm/h) were also noted. Aminotransferase, albumin, creatine kinase, b-type natriuretic peptide, and coagulation tests were normal.

The child was treated with antibiotics (cefotaxime and sulbactam, 100 mg/kg/d). To better screen for infectious diseases, autoimmune rheumatic diseases, and blood disorders, further laboratory tests were carried out. Cultures for blood, urine, and stool were negative. Acute infections of common respiratory tract pathogens, viruses, *Mycobacterium tuberculosis*, and fungi were excluded (Table 1). Assays of circulating cytokines showed a mildly raised IL-6 level. Lymphocyte subset analysis and levels of immunoglobulin and complement were both normal, indicating that the patient had normal immunity. In addition, autoantibodies for systemic autoimmune diseases were all negative. Bone marrow aspiration was performed, and the pathological findings showed hyperplasia of granulocytes, decreased erythroid hyperplasia, and a significant increase of platelets, which did not indicate any hematologic disease. In terms of imaging tests, renal ultrasound showed normal kidney, ureters, and bladder structures. An echocardiography examination showed mild mitral and tricuspid regurgitation with no dilation of the coronary artery.

**Table 1:** Etiology examinations of the patient.

**Etiology examinations**	**Test details**	**Results**
- Epstein-Barr virus	Antibody test	Negative
- The nine pathogens of respiratory tract infection (IgM antibodies)	*Legionella pneumophila* serogroup 1, *Mycoplasma pneumoniae, Coxiella burnetii, Chlamydia pneumoniae*, adenovirus, respiratory syncytial virus, influenza A virus, influenza B virus, parainfluenza virus type 1/2/3	All negative
- Measles	Antibody test for IgM	Positive^*^
- Enterovirus (IgM antibodies)	Echovirus Coxsackievirus A16 Coxsackievirus B	All negative
- Varicella-zoster virus	Antibody test for IgM	Negative
- SARS-CoV-2	Antibody test	IgG (–), IgM (–)
	Nucleic acid	Negative
- TORCH	Antibody test of IgM	All negative
- *Treponema pallidum*	Antibody test	Negative
- Parvovirus B19	Antibody test	Negative
- *Mycobacterium tuberculosis*	T-SPOT.TB test	Negative
- Fungi	β-D-Glucan assay	Both negative
	Galactomannan assay	

TORCH stands for toxoplasmosis, rubella, cytomegalovirus and herpes simplex virus.

* The boy had been vaccinated against measles, mumps, and rubella a month before the onset. The positive of anti-measles IgM was point to post-vaccination response.

The diagnosis of incomplete KD was initially considered depending on the duration of the fever, clinical features, the ineffectiveness of antibiotic treatment, and laboratory results before and after admission (9). Based on the diagnosis, high-dose IVIG (2 g/kg) and aspirin (5 mg/kg/day) were administered on day 4 post-admission. Thirty-six hours after the first infusion of high-dose IVIG, the boy's fever was persistent and a repeated infusion of IVIG (2 g/kg) was given. However, two doses of IVIG were still unable to relieve the fever. In addition, the hemoglobin level of the patient declined to 7.6 g/dL, and the platelet count rose to 885 × 10^9^/L, while the albumin level dropped to 28.1 g/L, which indicated resistance to IVIG. Then, corticosteroid (intravenous methylprednisolone, 2 mg/kg/day) was added as adjunctive therapy for KD. The antiplatelet medicine clopidogrel was also provided to prevent blood clots, and human albumin was given to increase the albumin level of the patient. After the aforementioned treatments were given, the patient's fever subsided, and periungual desquamation of fingers and toes occurred simultaneously. Repeated FBC testing showed a WBC count of 8.29 × 10^9^/L, and the CRP level decreased to normal. Following 4 days of intravenous methylprednisolone, steroid therapy was continued with oral prednisone, starting at a dose of 2 mg/kg/day, and tapering in the next few days.

**Figure 1:**
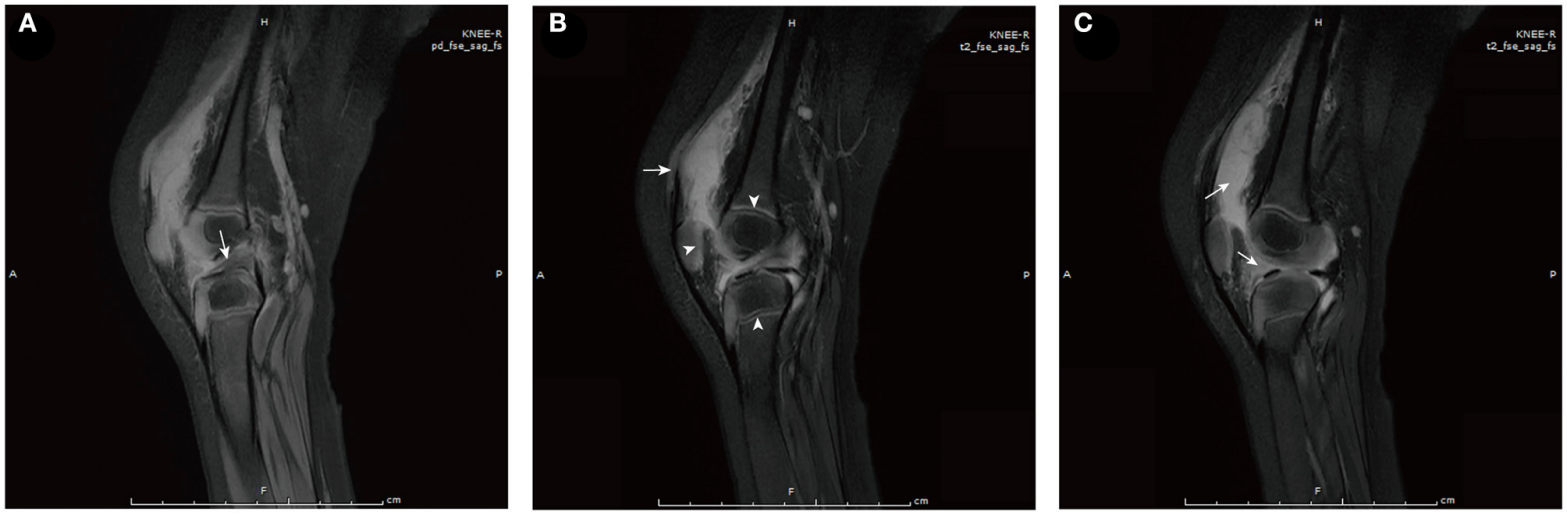
MRI of the right knee [proton density-weighted **(A)** and T2-weighted **(B, C)**]. **(A)** Edema of the anterior cruciate ligament (arrow). **(B)** Soft tissue around the lower segment of the femur was swollen (arrow). Arrowheads from left to right show abnormal signals in the patella, epiphysis of the femur, and epiphysis of the tibia successively, which may have been caused by infectious disease. **(C)** Massive effusion is observed in the suprapatellar capsule (left arrow) and joint cavity (right arrow).

**Figure 2:**
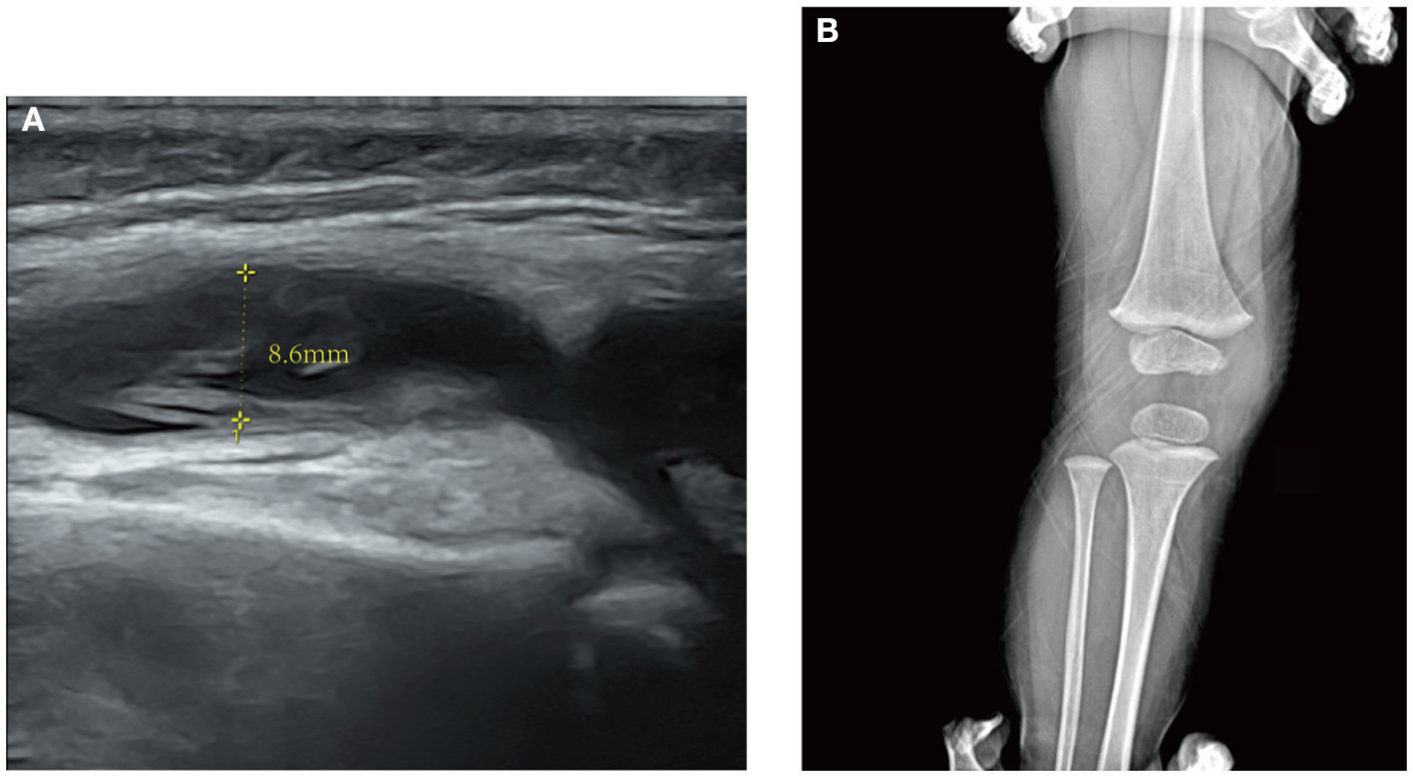
**(A)** Ultrasound of the right knee. An anechoic zone with an anteroposterior diameter of 8.6 mm is seen in the suprapatellar capsule of the joint suggesting effusion in the suprapatellar bursa. **(B)** X-ray of the right knee in a half-year follow-up of the patient after discharge. No obvious abnormality is found.

On the 5th day after the fever retreat, the boy developed slight swelling of the right knee with decreased joint mobility and a palpable skin temperature increase. MRI (Figure 1) and ultrasound (Figure 2A) tests were then performed to evaluate the condition of the right knee. The results demonstrated that infectious lesions existed in the right knee joint and its surrounding soft tissue, and there was massive effusion in the suprapatellar capsule and joint cavity. A final diagnosis of SA was considered in combination with the laboratory results (high CRP and ESR levels), signs, and imaging findings of the right knee (10). Thereafter, empiric antibiotics therapy was adjusted and teicoplanin was used to enhance the coverage of gram-positive bacteria.

Four days after the antibiotic adjustment, the patient developed a low-grade fever again. FBC testing showed a WBC count of 16.67 × 10^9^/L with a neutrophil ratio of 36.4%. The CRP level increased once more to 41.69 mg/L, which indicated the ineffectiveness of antibiotic therapy. To better eradicate the infection and to obtain samples for culture and pathology tests, the boy was then transferred to the pediatric surgery department for subsequent treatment. Soon after, arthroscopy-assisted synovial debridement and joint cavity drainage of the right knee were performed. Samples collected during the surgery were sent for microbiological and pathological analyses. The synovial fluid culture was negative for bacteria. The pathological findings of the synovial tissue showed a large amount of neutrophil exudate and small pieces of inflammatory granulation tissue, which were consistent with the changes in purulent inflammation.

**Figure 3:**
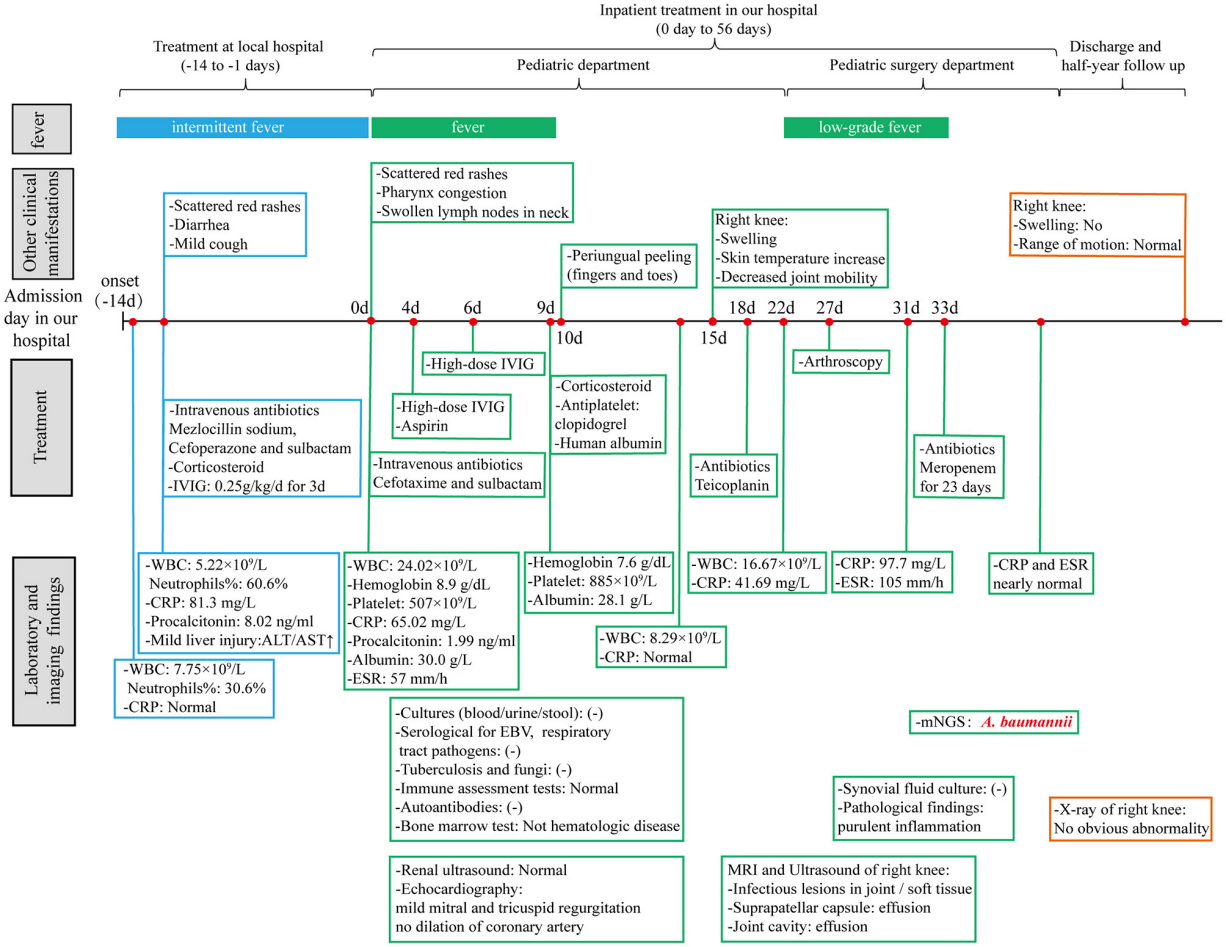
A timeline with relevant data from the patient's episode of care. WBC, white blood cell; CRP, C-reactive protein; ALT, alanine aminotransferase; AST, aspartate aminotransferase; IVIG, intravenous immunoglobulin; ESR, erythrocyte sedimentation rate; EBV, Epstein-Barr virus; mNGS, metagenomics next-generation sequencing.

However, after surgery, the patient still had an intermittent low fever. The levels of CRP and ESR continued to rise to 97.7 mg/L and 105 mm/h, respectively. To determine the organisms involved and to carry out targeted treatment, metagenomics next-generation sequencing (mNGS, using the BGISEQ-50/MGISEQ-2000 Sequencing Platform, BGI/MGI Tech, Shenzhen, China) of joint draining fluid for pathogen detection was done. The result showed that *A. baumannii* was positive with a stringent mapped reads number of 14,209. In addition, bacterial antibiotic resistance genes detection showed that the resistance genes of β-lactamase (CTX-M1), metallo-β-lactamase (IMP, VIM, NDM, SIM, DIM), and carbapenemase (KPC) were negative. Thereafter, meropenem (started at 60 mg/kg/day and tapering) was added immediately to strengthen antibiotics treatment. After antibiotic adjustment, his fever was relieved completely, and the WBC count and CRP levels gradually returned to normal. A rehabilitation approach was provided subsequently to improve his knee extension. The patient eventually recovered after 23 days of treatment and was discharged from the hospital. Thereafter, the boy received rehabilitation training outside the hospital. In the half-year follow-up of the patient, the range of motion of the right knee had returned to normal, and no obvious abnormality was found in the repeated X-ray examination (Figure 2B). A timeline, with relevant data from the patient's episode of care, is shown in Figure 3.

## Discussion

In the present case, the patient went through a complex and tortuous process of diagnosis and treatment for over 2 months. The initial diagnosis of incomplete KD (IVIG resistant) was established based on the clinical manifestations (fever lasted for over 5 days, the appearance of rashes, cervical lymphadenopathy, and periungual desquamation), laboratory results (high CRP and ESR, leukocytosis, anemia, hypoalbuminemia, and thrombocytosis), and response to treatment (9). Differential diagnosis was performed simultaneously. Viruses (such as respiratory virus and enterovirus), *Mycobacterium tuberculosis*, and fungi infections were excluded based on etiology tests. The only positive serological result was measles-IgM. However, the boy had been vaccinated against measles, mumps, and rubella a month before the onset. Combined with the result of significantly elevated CRP and ESR, the positivity of IgM indicates post-vaccination response. Systemic juvenile idiopathic arthritis was also ruled out based on atypical rash and periungual desquamation, which was further verified by later purulent joint changes. Moreover, systemic autoimmune and hematologic diseases were excluded based on autoantibodies detection and bone marrow cytology. As the disease progressed, the child subsequently developed joint symptoms in the right knee. The final diagnosis of SA was made in combination with imaging and laboratory results (10). After arthroscopic surgery, *A. baumannii* was finally identified in the mNGS test of the joint draining fluid.

*A. baumannii* is a gram-negative coccobacillus that can survive for long periods in the environment and has remarkable abilities to acquire various resistance determinants (2), primarily causing hospital-acquired infections. The WHO declared that *A. baumannii* is one of the six most serious organisms with multidrug resistance and virulence (11). Although regarded as a low-grade pathogen, *A. baumannii* infection is usually associated with high mortality (2), especially affecting those who are immunocompromised and have a long history of hospitalization (12). The respiratory tract, bloodstream, skin and soft tissue, urinary tract, and central nervous system are frequently infected by *A. baumannii* (2, 13). However, *A. baumannii*-associated SA is rarely reported (6–8). One case was reported in a patient with gout, who was treated with acupuncture before fever and joint symptoms of the knee (6). The initial bacterial culture of joint purulent discharge was positive for *Staphylococcus aureus*. After joint draining and administration of broad-spectrum antibiotics, the patient's symptoms were relieved transiently and reoccurred subsequently. Repeated bacterial culture of synovial fluid suggested *A. baumannii* infection. The other two cases both described patients of SA with mixed bacterial infection, including *A. baumannii*. One of the patients underwent arthroscopic surgery 5 months before the onset of SA (7). The other patient had a purulent infection following trauma to the right knee (8). In these three described cases, all patients were adults, and they had a knee injury or exposure prior to onset, providing more favorable conditions for the invasion of *A. baumannii*. Whereas, the present manuscript describes a rare case of *A. baumannii* SA in an infant who has not undergone invasive surgery or had injury in the knee.

Septic arthritis is a synovial joint infection generally caused by bacteria (14). In children, boys aged below 2–4 years are more frequently affected (15, 16). Hematogenous spread is a more common route of infection than direct inoculation and nearby spread of osteomyelitis (10, 17). Pathogens may vary according to the child's age, presence of comorbidity or immunodeficiency, vaccination status, and socioeconomic factors. *Staphylococcus aureus* is the primary causative organism (17). Besides, *Kingella kingae*, Group A *Streptococcus, Streptococcus pneumoniae*, and *Haemophilus influenzae* type b are the main causes in children aged from 3 months to 5 years, among which the last two usually infect unimmunized children. However, *A. baumannii* infection is seldom reported. In the present case, the boy had no puncture or open trauma of the right knee, and MRI images showed no nearby osteomyelitis, suggesting hematogenous spread as the most likely route of infection. Whereas, the patients' immune assessment tests showed normal cellular and humoral immunity. The potential risk factors were long history of hospitalization, administration of intravenous broad-spectrum antibiotics, and short-term corticosteroid therapy (15). Combined with the characteristics of *A. baumannii*, it is speculated that the patient had a hospital-acquired infection and the entry route of *A. baumannii* may have been through the respiratory tract or venipuncture.

Since SA can lead to significant acute and chronic disability, early diagnosis and initiation of treatment are critical (14). Considering the common causative pathogens, empiric treatment primarily targets *S. aureus* (10). In cases when empiric antibiotic therapy is ineffective, the identification of microorganisms may be helpful to choose more targeted antibiotics. However, the sensitivity of blood and/or synovial fluid cultures is quite low in children (positive in only 20–70% of cases) (10, 16), and cultures may be negative even in the face of severe infection. Fortunately, due to improvements in diagnostic technology, such as nucleic acid amplification, more pathogens can now be identified (15). Traditional molecular biology techniques such as PCR can involve large numbers of individual tests for targeted organisms but may still miss rare and emerging pathogens (18). In addition, primers used in PCR may mismatch the microbial strain, which may reduce the sensitivity of the assay. Nevertheless, with the development of sequencing technology, high-throughput and low-cost next-generation sequencing has emerged, among which mNGS has been most widely studied (19). It can obtain the sequence information of microbial nucleic acid fragments in a single run and identify more potential pathogens than conventional methods without sacrificing sensitivity, and is becoming an increasingly available method to detect pathogens in suspected infection cases (20). When polymicrobial samples are involved, quantitative or semiquantitative data on the concentration of organisms can be obtained through the counting of sequenced reads. Besides, it can also provide additional genomic information for evolutionary tracing, strain identification, and drug resistance prediction (18). In the present report, as a rare pathogen, *A. baumannii* was established by mNGS testing, and the result of drug resistance genes detection excluded the existence of some resistance genes, which helped us to better treat the boy and reduce the occurrence of sequelae to a large extent.

## Conclusion

Although uncommon, *A. baumannii* can also lead to SA in infants, especially those with a long history of hospitalization and antibiotics administration. Due to the insidious onset and atypical symptoms, bone and joint examination should be done in febrile infants to pay attention to the existence of SA. When microbial culture fails to obtain positive results, mNGS can be used to increase the early pathogen detection rate, reduce the unnecessary use of broad-spectrum antibiotics, and ultimately improve the prognosis of the disease.

## Data availability statement

The original contributions presented in the study are included in the article/supplementary material, further inquiries can be directed to the corresponding author.

## Ethics statement

Written informed consent was obtained from the minor(s)' legal guardian/next of kin for the publication of any potentially identifiable images or data included in this article.

## Author contributions

YL wrote the first draft of the manuscript. JX and XL contributed to manuscript revision. All authors participated in the patient's care and read and approved the submitted version.

## References

[1] Lee CR Lee JH Park M Park KS Bae IK Kim YB. Biology of *Acinetobacter baumannii*: pathogenesis, antibiotic resistance mechanisms, and prospective treatment options. Front Cell Infect Microbiol. (2017) 7:55. 10.3389/fcimb.2017.00055 28348979 PMC5346588

[2] Ibrahim S Al-Saryi N Al-Kadmy IMS Aziz SN. Multidrug-resistant *Acinetobacter baumannii* as an emerging concern in hospitals. Mol Biol Rep. (2021) 48:6987–98. 10.1007/s11033-021-06690-6 34460060 PMC8403534

[3] Liu X Zhou H Shu S Li G Fang F. Successful treatment with tigecycline of severe pandrug-resistant *Acinetobacter baumannii* pneumonia complicated with a diaphragmatic hernia: a case report. Ann. Palliat Med. (2022) 12:193–9. 10.21037/apm-22-900 36408560

[4] Nasr P. Genetics, epidemiology, and clinical manifestations of multidrug-resistant *Acinetobacter baumannii*. J Hosp Infect. (2020) 104:4–11. 10.1016/j.jhin.2019.09.021 31589900

[5] Garnacho-Montero J Timsit JF. Managing *Acinetobacter baumannii* infections. Curr Opin Infect Dis. (2019) 32:69–76. 10.1097/QCO.0000000000000518 30520737

[6] Duan X Yang L Xia P. Septic arthritis of the knee caused by antibiotic-resistant *Acinetobacter baumannii* in a gout patient: a rare case report. Arch Orthop Trauma Surg. (2010) 130:381–4. 10.1007/s00402-009-0958-x 19707778

[7] Marchi MF Gomes RS. Septic oligoarthritis caused by *Klebsiella pneumoniae* and *Acinetobacter baumannii*. J Rheumatol. (2013) 40:1239–40. 10.3899/jrheum.130117 23818730

[8] Chiu LQ Wang W. A case of unusual Gram-negative bacilli septic arthritis in an immunocompetent patient. Singapore Med J. (2013) 54:e164–8. 10.11622/smedj.2013162 24005465

[9] McCrindle BW Rowley AH Newburger JW Burns JC Bolger AF Gewitz M. Diagnosis, treatment, and long-term management of kawasaki disease: a scientific statement for health professionals from the American Heart Association. Circulation. (2017) 135:e927–99. 10.1161/CIR.0000000000000484 28356445

[10] Paakkonen M. Septic arthritis in children: diagnosis and treatment. Pediatric Health Med Ther. (2017) 8:65–8. 10.2147/PHMT.S115429 29388627 PMC5774603

[11] Boucher HW Talbot GH Bradley JS Edwards JE Gilbert D Rice LB. Bad bugs, no drugs: no ESKAPE! An update from the Infectious Diseases Society of America. Clin Infect Dis. (2009) 48:1–12. 10.1086/595011 19035777

[12] Howard A O'Donoghue M Feeney A Sleator RD. *Acinetobacter baumannii*: an emerging opportunistic pathogen. Virulence. (2012) 3:243–50. 10.4161/viru.19700 22546906 PMC3442836

[13] Ayoub Moubareck C Hammoudi Halat D. Insights into *Acinetobacter baumannii*: a review of microbiological, virulence, and resistance traits in a threatening nosocomial pathogen. Antibiotics. (2020) 9:119. 10.3390/antibiotics9030119 32178356 PMC7148516

[14] Montgomery NI Epps HR. Pediatric septic arthritis. Orthop Clin North Am. (2017) 48:209–16. 10.1016/j.ocl.2016.12.008 28336043

[15] Swarup I Meza BC Weltsch D Jina AA Lawrence JT Baldwin KD. Septic arthritis of the knee in children: a critical analysis review. JBJS Rev. (2020) 8:e0069. 10.2106/JBJS.RVW.19.00069 32105243

[16] Cohen E Katz T Rahamim E Bulkowstein S Weisel Y Leibovitz R. Septic arthritis in children: updated epidemiologic, microbiologic, clinical and therapeutic correlations. Pediatr Neonatol. (2020) 61:325–30. 10.1016/j.pedneo.2020.02.006 32184066

[17] Erkilinc M Gilmore A Weber M Mistovich RJ. Current concepts in pediatric septic arthritis. J Am Acad Orthop Surg. (2021) 29:196–206. 10.5435/JAAOS-D-20-00835 33273402

[18] Gu W Miller S Chiu CY. Clinical metagenomic next-generation sequencing for pathogen detection. Annu Rev Pathol. (2019) 14:319–38. 10.1146/annurev-pathmechdis-012418-012751 30355154 PMC6345613

[19] Duan H Li X Mei A Li P Liu Y Li X. The diagnostic value of metagenomic next rectanglegeneration sequencing in infectious diseases. BMC Infect Dis. (2021) 21:62. 10.1186/s12879-020-05746-5 33435894 PMC7805029

[20] Miller S Chiu C. The role of metagenomics and next-generation sequencing in infectious disease diagnosis. Clin Chem. (2021) 68:115–24. 10.1093/clinchem/hvab173 34969106

